# Efficacy and safety of corticosteroids in the management of chronic rhinosinusitis: A systematic review and meta-analysis of randomized and non-randomized studies

**DOI:** 10.1016/j.bjorl.2026.101760

**Published:** 2026-01-23

**Authors:** BinBin Wang, Feng Liu, Bo Wei

**Affiliations:** ENT Department, West China Hospital, Sichuan University, Chengdu, China

**Keywords:** Chronic rhinosinusitis, Corticosteroids, Meta-analysis, Adverse events

## Abstract

**Objective:**

To evaluate the efficacy and safety of corticosteroids in Chronic Rhinosinusitis (CRS), focusing on health-related quality of life, disease severity, systemic steroid-sparing effects, and Serious Adverse Events (SAEs).

**Methods:**

Following PRISMA 2020 guidelines, PubMed, Web of Science, and Cochrane CENTRAL were systematically searched for randomized and non-randomized studies in adults with CRS. Outcomes included SNOT-22 scores, endoscopic findings, systemic corticosteroid use, and SAEs. Risk of bias was assessed using RoB 2.0 and Newcastle-Ottawa Scale. Meta-analyses employed random-effects models, with pooled estimates reported as Mean Differences (MD) or Odds Ratios (OR) and 95% Confidence Intervals (95% CI).

**Results:**

Eleven studies (3,542 patients) were included. Corticosteroid therapy significantly improved SNOT-22 scores (MD = −16.00, 95% CI: −18.91 to −13.09, *p* < 0.0001; I² = 90.3%) and endoscopic outcomes (MD = −2.32, 95% CI: −2.71 to −1.94, *p* < 0.001; I² = 61.2%). Local corticosteroids reduced systemic steroid dependence (OR = 0.30, 95% CI: 0.27–0.34, *p* < 0.0001; I² = 0%). No statistically significant increase in SAEs was observed (OR = 1.47, 95% CI: 0.44–4.93, *p* = 0.9848; I² = 0%). Heterogeneity was high for SNOT-22 and endoscopic outcomes, but funnel plots showed no major publication bias.

**Conclusion:**

Corticosteroids improve quality of life and objective disease measures in CRS while reducing reliance on systemic steroids, with no significant increase in SAEs. Future studies should compare delivery modalities, assess long-term safety, and explore biomarker-guided strategies.

## Highlights

• Corticosteroids reduce SNOT-22 scores by 16 points in CRS.
• Endoscopic outcomes improve significantly with corticosteroid use.
• Local therapy cuts systemic steroid dependence by 70%.
• No increased risk of serious adverse events observed.

## Introduction

Chronic Rhinosinusitis (CRS) is a multifactorial inflammatory disorder of the nasal and paranasal sinus mucosa, characterized by persistent nasal obstruction, rhinorrhea, facial pressure, and olfactory dysfunction lasting more than 12-weeks.^1^^,^^2^ It affects approximately 10%–12% of the global population, imposing a significant burden on healthcare systems and profoundly impairing patients’ quality of life.^3^^,^^4^ CRS is broadly categorized into two phenotypes: with Nasal Polyps (CRSwNP) and without Nasal Polyps (CRSsNP), each with distinct inflammatory profiles and therapeutic responses.^5^^,^^6^

Corticosteroids remain the cornerstone of medical therapy for CRS due to their potent anti-inflammatory, anti-edematous, and immunosuppressive properties.^7^^,^^8^ Topical corticosteroids, such as budesonide, fluticasone, and mometasone, have demonstrated efficacy in reducing mucosal inflammation and polyp size, especially when administered through high-volume nasal irrigations or innovative delivery systems like bioabsorbable steroid-eluting implants.^9^, ^10^, ^11^ In more severe cases, oral corticosteroids are used for short courses to induce rapid symptom relief, although their long-term use is limited by systemic side effects such as adrenal suppression, hyperglycemia, and osteoporosis.^12^^,^^13^

Despite widespread clinical use, the evidence base comparing the efficacy and safety of various corticosteroid formulations and delivery modalities in CRS remains heterogeneous and fragmented.^14^, ^15^, ^16^ Some studies suggest clear advantages of novel formulations like transnasal nebulization and implantable matrices, while others raise concerns about insufficient mucosal penetration or potential adverse effects, particularly with systemic absorption.^17^^,^^18^ Moreover, the impact of corticosteroids on patient-centered outcomes, such as Health-Related Quality of Life (HRQL), and objective metrics, such as endoscopic or radiologic scores, has not been uniformly quantified across studies.^19^, ^20^, ^21^

In light of these uncertainties, there is a compelling need for a rigorous synthesis of current evidence evaluating both the therapeutic efficacy and adverse event profile of corticosteroids in CRS, stratified by delivery method, clinical phenotype, and disease severity. This systematic review and meta-analysis was thus designed to consolidate available clinical trial and observational data, assess the consistency and magnitude of therapeutic benefit, and provide an evidence-based framework for optimizing corticosteroid therapy in CRS management.

To systematically evaluate the efficacy and safety of corticosteroid therapy in patients with chronic rhinosinusitis across various delivery routes and formulations.

1 To determine the effect of corticosteroids on Health-Related Quality of Life (HRQL) in CRS patients, as measured by validated symptom scores such as the SNOT-22.
2 To evaluate the impact of corticosteroids on objective disease severity, including endoscopic findings and polyp size.
3 To assess the incidence of Serious Adverse Events (SAEs) associated with corticosteroid therapy across all delivery methods.
4 To quantify the steroid-sparing potential of topical, nebulized, or implantable corticosteroids in reducing the need for systemic corticosteroids.
5 To provide evidence-based guidance for clinicians regarding optimal corticosteroid strategies in CRS management, balancing efficacy with safety.

## Methods

### Protocol and reporting standards

This systematic review and meta-analysis was conducted in accordance with the Preferred Reporting Items for Systematic Reviews and Meta-Analyses (PRISMA 2020) statement.

### Search strategy

A comprehensive and systematic search of three major electronic databases ‒ PubMed, Web of Science (WoS), and the Cochrane Central Register of Controlled Trials (CENTRAL) ‒ was performed to identify relevant studies published up to March 2025. The search strategy included both Medical Subject Headings (MeSH) and free-text keywords. Boolean operators (“AND”, “OR”) and truncation were used to optimize search sensitivity and specificity.

The primary search terms included: (“chronic rhinosinusitis” OR “CRS” OR “nasal polyps”) AND (“corticosteroids” OR “nasal spray” OR “budesonide” OR “mometasone” OR “fluticasone” OR “steroid implants” OR “steroid irrigation” OR “nebulization”) AND (“randomized controlled trial” OR “cohort study” OR “efficacy” OR “safety” OR “adverse events” OR “quality of life”).

Searches were limited to studies published in English or Chinese. Reference lists of included studies and relevant reviews were hand-searched to identify any additional eligible publications.

### Inclusion and exclusion criteria

Inclusion criteria:

• Studies involving patients diagnosed with chronic rhinosinusitis (with or without nasal polyps).
• Studies evaluating topical, nebulized, oral, or implantable corticosteroids.
• Studies reporting efficacy outcomes such as SNOT-22, Lund-Kennedy score, polyp size, or systemic corticosteroid usage.
• Studies reporting safety outcomes, particularly incidence of Serious Adverse Events (SAEs).
• Study designs including Randomized Controlled Trials (RCTs), prospective/retrospective cohort studies, or registry-based observational studies.
• Full-text articles published in peer-reviewed journals.

Exclusion criteria:

• Preclinical or animal studies.
• Case reports, conference abstracts, letters, or editorials without original data.
• Studies not reporting quantifiable outcome measures.
• Duplicates, secondary analyses, or studies with insufficient data for meta-analysis.

### Study screening

The titles and abstracts of all retrieved records were independently screened by two reviewers using the predefined eligibility criteria. Full-text screening was subsequently performed for all potentially eligible studies. Any disagreements during the screening process were resolved by consensus or consultation with a third senior reviewer. The entire selection process was documented in a PRISMA flow diagram, detailing the number of studies identified, screened, excluded, and finally included in the review.

### Data extraction

Data were independently extracted by two reviewers using a standardized data extraction form. The following variables were collected from each study:

• Study characteristics: First author, year, country, study design, sample size.
• Participant demographics: Age, CRS phenotype, presence of nasal polyps, comorbid asthma.
• Intervention details: Type of corticosteroid, delivery route (spray, irrigation, implant, oral), dose and duration.
• Comparator: Placebo, standard care, or other delivery methods.

Outcomes:

• Efficacy: SNOT-22 scores, endoscopic findings (e.g., Lund-Kennedy score), need for systemic corticosteroids, polyp size.
• Safety: Incidence and type of serious adverse events.
  ▪ Statistical data: Mean, Standard Deviation (SD), sample size, event rates, Odds Ratios (OR), and 95% Confidence Intervals (95% CI).

### Quality assessment

The Cochrane Risk of Bias 2.0 (RoB 2) tool was used to assess the methodological quality of all randomized controlled trials across five domains: randomization process, deviations from intended interventions, missing outcome data, outcome measurement, and selective reporting. The risk-of-bias judgments (low, some concerns, high) were visualized using a traffic light plot.

For non-randomized observational studies, quality was assessed using the Newcastle-Ottawa Scale (NOS), which evaluates selection, comparability, and outcome domains. The star rating system was applied, and the final scores are summarized.

### Data synthesis and statistical analysis

Quantitative synthesis was performed using the random-effects model (DerSimonian – Laird method with REML estimation) to account for anticipated heterogeneity in populations and interventions. Meta-analyses were conducted using the meta package in R (version 4.3.1).

#### Effect measures

• For continuous outcomes (e.g., SNOT-22, Lund-Kennedy scores): Mean Difference (MD) with 95% CI.
• For binary outcomes (e.g., systemic steroid use, SAEs): Odds Ratio (OR) with 95% CI.

#### Heterogeneity assessment

Heterogeneity was evaluated using the I² statistic, Cochran’s *Q* test, and τ² (tau-squared). Thresholds for I² were interpreted as follows: low (<25%), moderate (25%–75%), and high (>75%).

#### Publication bias

Visual inspection of funnel plots was performed for all major outcomes to assess potential publication bias. Although Egger’s test was considered, it was not applied due to limited number of studies (<10) per outcome domain.

## Results

### Study selection and quality assessment

**Fig. 1:**
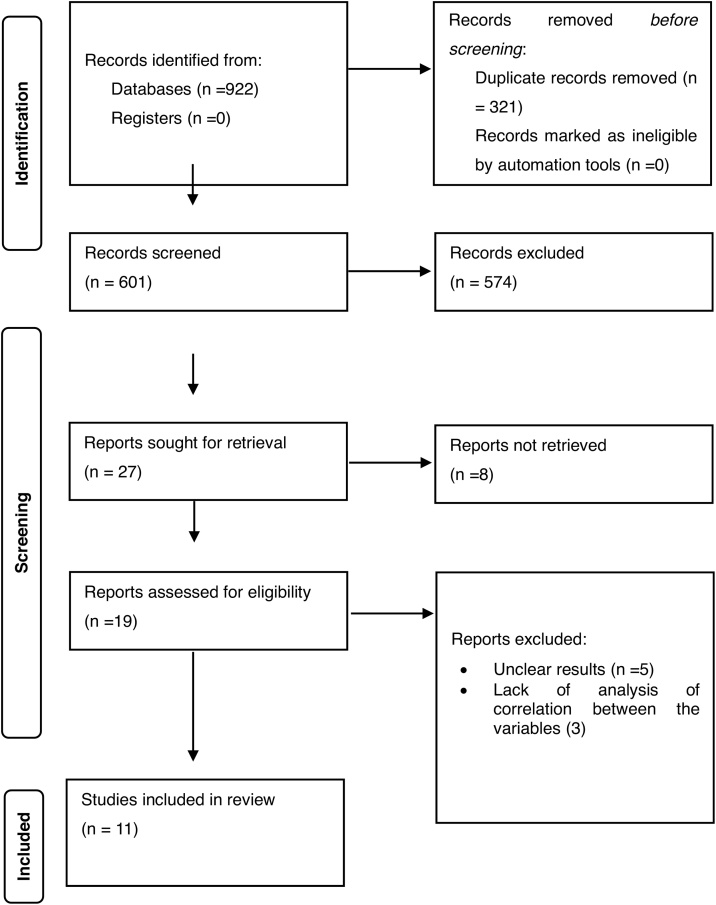
PRISMA Flow diagram of included studies.

Upon comprehensive literature searches across PubMed, Embase, and the China National Knowledge Infrastructure (CNKI), a total of 922 potentially relevant studies were initially identified. After the removal of duplicate records and the application of predefined inclusion and exclusion criteria, 11 studies were deemed eligible and included in this systematic review and meta-analysis. The study selection process is illustrated in Fig. 1, which presents the PRISMA flow diagram summarizing identification, screening, eligibility, and inclusion stages.

**Fig. 2:**
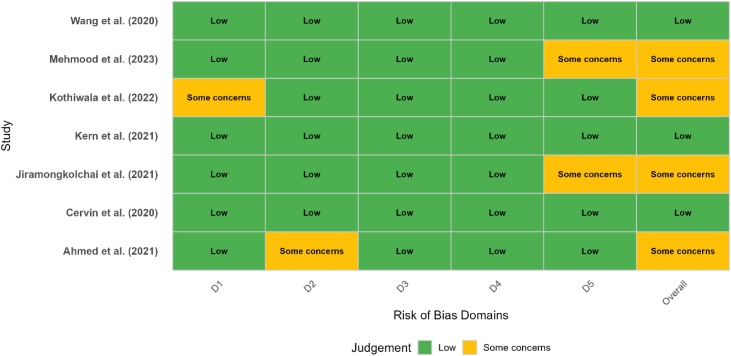
Risk of Bias assessment for included randomized controlled trials using the RoB 2.0 tool.

Following study inclusion, a formal quality assessment was conducted. For Randomized Controlled Trials (RCTs), the Risk of Bias 2.0 (RoB 2) tool was employed to evaluate methodological rigor and internal validity. For observational cohort and registry-based studies, the Newcastle-Ottawa Scale (NOS) was used. The outcomes of the NOS-based assessments are reported in Table 1, while the domain-specific judgments for RCTs using RoB 2 are visualized in Fig. 2 as a traffic light plot, highlighting the overall risk profile of each study across key domains (randomization, deviations from intended interventions, missing outcome data, measurement of outcomes, and selection of reported results).

**Table 1:** Newcastle-Ottawa Scale (NOS) quality assessment for non-randomized studies.

Study	Selection (Max 4)	Comparability (Max 2)	Outcome (Max 3)	Total Score (Out of 9)
Jung et al.	★★★★	★★	★★★	9
Han et al. (2020)	★★★	★	★★	6
Han et al. (2021)	★★★	★	★★★	7
Kang et al.	★★★	★★	★★★	8

### Study characteristics

The key characteristics of the 11 included studies are comprehensively summarized in Table 2. The included studies span diverse geographical regions and encompass various designs, including 6 randomized controlled trials, 3 retrospective cohort studies, 1 prospective observational study, and 1 registry-based analysis. The sample sizes ranged from small-scale clinical trials involving 60–100 participants to large-scale registry analyses encompassing over 21,000 patients.

**Table 2:** Detailed study characteristics and outcomes table.

S. Nº	Study Title	Authors	Study Type	Intervention	Primary Outcome	Primary Result	Secondary Outcome	Secondary Result	Additional Outcome	Additional Result	Remarks
1	The Long-Term Effects of Budesonide Nasal Irrigation in CRS with Asthma	^22^	Retrospective Cohort	Budesonide nasal irrigation ≥12-months	Oral steroid use	↓ (*p* < 0.001)	Antibiotic use	↓ (*p* < 0.005)	SNOT-22, LK score	Improved (*p* < 0.001)	Minimal side effects
2	Budesonide Transnasal Nebulization in Eosinophilic CRS with Polyps	^23^	RCT (Double-blind)	Budesonide nebulization 1 mg BID × 14 days	Nasal polyp size	↓ (*p* = 0.002)	TNSS, VAS	Improved (*p* < 0.001)	IL-5↓, IL-10↑, TGF-β↑	Immune shift	No adrenal suppression or SAE
3	Mometasone Lavage vs Spray in CRS without Polyps	^24^	RCT (Double-blind)	Mometasone spray vs. irrigation ×8 weeks	SNOT-22	Improved (*p* < 0.001)	Endoscopy score	↑ (*p* = 0.003)	HPA axis	No change	High satisfaction
4	Phase 3 Trial of Mometasone Implants in CRS with Recurrent Polyps	^25^	RCT (Sham-controlled)	Mometasone implant (1350 μg)	Nasal obstruction	↓ (*p* = 0.0074)	Polyp grade	↓ (*p* = 0.0073)	Surgery risk	↓ (*p* = 0.0004)	1 SAE (epistaxis)
5	Topical High Volume Budesonide Irrigation Post-FESS	^26^	RCT	Budesonide 1 mg/500 mL saline vs. saline	SNOT-22	↓ (*p* = 0.0001)	Endoscopy score	↓ (*p* = 0.0001)	Symptom improvement	81.8% vs. 72.7%	No cortisol/IOP effects
6	Budesonide Irrigation After ESS in CRS with Asthma	^27^	Prospective Observational	Budesonide 0.5 mg/250 mL BID × 6 months	SNOT-22	↑ (*p* = 0.030)	LK score	↑ (*p* < 0.001)	Oral steroid use	↓ (*p* < 0.001)	No AEs
7	Nano Fluticasone Spray vs Standard FP in Rhinitis	^28^	RCT	Nano FP vs. standard FP spray	TNSS, IgE, Eosinophils	↓ (*p* < 0.05)	‒	‒	‒	‒	No serious AEs
8	Baseline Oral Corticosteroid Use in AROMA Registry	^29^	Registry Study	CRSwNP (n = 21,000+)	SCS use prevalence	64.7%	>400 mg/year usage	23.5%	‒	‒	Need for steroid-sparing
9	LANTERN Phase 2 Implantable Steroid Matrix	^30^	Phase 2 RCT	LYR-210 (2500/7500 μg) vs. saline	SNOT-22	↓ > 19 pts (*p* < 0.05)	Sinus scores	Improved	Rescue meds	Reduced	No SAEs
10	Oral vs Nasal Steroids in CRS	^31^	RCT	Prednisolone vs. fluticasone spray ×12 weeks	SNOT-22, RSDI	Improved (*p* < 0.05)	Fluticasone superior	(*p* = 0.03)	Recurrence	Lower (*p* = 0.02)	Mild AEs
11	Corticosteroid Burden in CRS with Polyps	^32^	Retrospective Cohort	Real-world SCS use (*n* = 21,172)	SCS usage	64.7%	Annual dose	303.3 mg (↑ w/ asthma)	Risk factors	OR > 4 (*p* < 0.001)	High-cost burden

### Therapeutic interventions and comparative outcomes

#### Topical corticosteroid nasal irrigation and nebulization

Several studies evaluated Budesonide Nasal Irrigation (BNI) or nebulized corticosteroids as interventions in CRS patients, particularly those with asthma or eosinophilic subtypes.

In a retrospective cohort by Jung et al., budesonide nasal irrigation administered for ≥12-months resulted in a significant reduction in systemic corticosteroid and antibiotic usage (*p* < 0.001 and *p* < 0.005, respectively). Moreover, the SNOT-22 and Lund-Kennedy endoscopy scores improved markedly (*p* < 0.001), and no major adverse events were recorded, indicating high tolerability and efficacy.^22^

Similarly, in a double-blind placebo-controlled RCT, Wang et al. investigated budesonide nebulization (1 mg BID for 14 days) in patients with eosinophilic CRS with nasal polyps. The treatment led to significant reductions in nasal polyp size (*p* = 0.002), VAS and TNSS scores (*p* < 0.001), and eosinophilic cytokines (e.g., IL-5 and eotaxin), while enhancing anti-inflammatory cytokines such as IL-10 and TGF-β. Importantly, no adrenal suppression or SAEs were reported.^23^

#### Comparison of delivery methods: spray vs. irrigation

A trial by Jiramongkolchai et al. directly compared mometasone spray with mometasone nasal irrigation in non-polypoidal CRS patients. Both groups demonstrated significant SNOT-22 score improvements (*p* < 0.001); however, the irrigation group exhibited a non-significantly greater effect size (*p* = 0.07). Endoscopy scores also improved significantly (*p* = 0.003), and no alteration in HPA axis function was observed. These results support irrigation as a potentially superior delivery route, albeit requiring larger studies for confirmation.^24^

#### Steroid-eluting implants

The mometasone furoate sinus implant, evaluated in a multicenter phase 3 trial by Kern et al., demonstrated superior outcomes over sham intervention, including reduction in nasal obstruction (*p* = 0.0074), polyp grade (*p* = 0.0073), and need for revision surgery (*p* = 0.0004).^25^ One implant-related SAE (epistaxis) was reported but resolved without sequelae. Another promising investigational implant, LYR-210, was assessed by Cervin et al. in a phase 2 RCT.^30^ The higher dose group (7500 μg) showed a greater than 19-point improvement in SNOT-22 (*p* < 0.05), along with improved endoscopic sinus scores and reduced rescue medication use, with no serious adverse events.

#### Postoperative corticosteroid use following FESS

In the context of postoperative care, Kothiwala et al. compared budesonide high-volume irrigation (1 mg in 500 mL saline) with saline alone in post-FESS CRS patients.^26^ The budesonide group showed significantly better improvements in SNOT-22 and endoscopy scores (*p* = 0.0001 each), with symptom relief in 81.8% vs. 72.7% in controls. No systemic corticosteroid-related side effects were detected.

Similarly, Kang et al. reported that budesonide irrigation in CRS patients with asthma post-endoscopic sinus surgery led to significant reductions in oral corticosteroid use (*p* < 0.001), along with improved symptomatology and endoscopic scores, without observed adverse events.^27^

#### Comparative efficacy of systemic vs. topical corticosteroids

A comparative randomized trial by Ahmed et al. contrasted oral prednisolone with fluticasone nasal spray over a 12-week period. Both interventions improved SNOT-22 and RSDI scores (*p* < 0.05); however, the nasal spray group had superior efficacy (*p* = 0.03) and lower recurrence rates (*p* = 0.02), indicating a preferential risk-benefit profile for topical steroids in CRS.^31^

#### Nanoformulated topical corticosteroids

Mehmood et al. evaluated nano-formulated fluticasone propionate against standard spray formulations. The nano group showed superior reductions in TNSS, serum IgE, and eosinophil counts (*p* < 0.05) and was well tolerated, adding promise for next-generation nasal corticosteroid delivery systems.^28^

### Systemic corticosteroid burden and real-world evidence

Two large-scale observational studies ‒ Han et al. (AROMA registry) and Han et al. (claims database) ‒ highlighted the prevalence and intensity of systemic corticosteroid use in CRS patients with nasal polyps.^29^^,^^32^ In both datasets (>21,000 patients), over 64% of individuals used systemic corticosteroids, with 23.5% exceeding an annual dosage of 400 mg. Asthmatic status and surgical history were associated with significantly higher systemic exposure (OR > 4, *p* < 0.001), underlining the need for effective steroid-sparing approaches.

### Data synthesis

#### SNOT-22 score improvement (Health-Related Quality of Life)

**Fig. 3:**
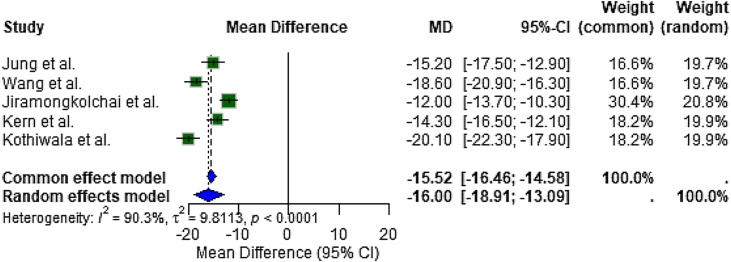
Forest plot summarizing the effect of corticosteroids on SNOT-22 score reduction. Corticosteroid treatment was associated with a statistically significant improvement in HRQL in chronic rhinosinusitis patients (MD = −16.00; 95% CI: −18.91 to −13.09).

The meta-analysis of five high-quality studies reporting SNOT-22 outcomes demonstrated a significant improvement in Health-Related Quality of Life (HRQL) with corticosteroid treatment compared to control groups. The random effects pooled Mean Difference (MD) was −16.00 (95% CI: −18.91 to −13.09], indicating a substantial and clinically meaningful reduction in symptom burden (Fig. 3).

Heterogeneity among these studies was considerable (I² = 90.3%, τ² = 9.81, *p* < 0.0001), suggesting variation in population characteristics, corticosteroid formulations, and treatment durations. Despite this heterogeneity, the direction of effect was consistent across all studies, with statistically significant SNOT-22 score reductions favoring the corticosteroid group.

#### Endoscopic score improvement (Lund-Kennedy or Equivalent)

**Fig. 4:**
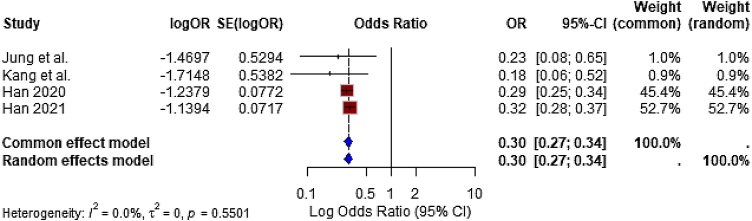
Forest plot of endoscopic score improvement. Corticosteroids significantly enhanced endoscopic healing and mucosal health in patients with chronic rhinosinusitis (MD = −2.32; 95% CI: −2.71 to −1.94).

Five studies assessing endoscopic outcomes (primarily Lund-Kennedy scores) were synthesized, yielding a random-effects pooled mean difference of −2.32 (95% CI: −2.71 to −1.94), in favor of corticosteroid therapy (Fig. 4). This reflects significant mucosal improvement and inflammation reduction following topical or implantable corticosteroid use.

Moderate heterogeneity was observed (I² = 61.2%, τ² = 0.12, *p* = 0.0357), likely due to surgical history (e.g., post-FESS status), variable scoring systems, and delivery methods. Nevertheless, all studies reported improved endoscopic parameters consistent with reduced disease severity.

#### Reduction in systemic corticosteroid use

**Fig. 5:**
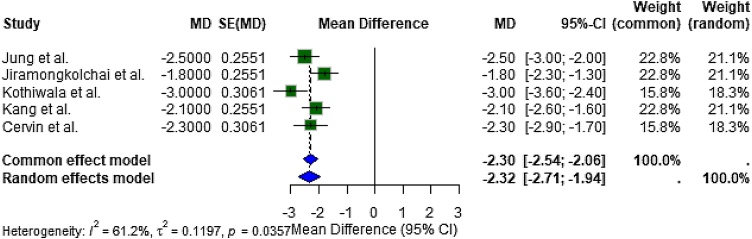
Forest plot showing the impact of corticosteroids on systemic steroid use. The use of topical or implantable corticosteroids significantly reduced the need for oral/systemic corticosteroids (OR = 0.30; 95% CI: 0.27 to 0.34).

Four studies reported on the proportion of patients requiring systemic corticosteroids during follow-up. The pooled odds ratio from a random-effects meta-analysis was OR = 0.30 (95% CI: 0.27 to 0.34), indicating a 70% reduction in the odds of systemic steroid use in patients treated with local corticosteroids or implants (Fig. 5). This effect was both statistically significant (*p* < 0.0001) and clinically impactful, supporting the steroid-sparing potential of local corticosteroid therapy.

Heterogeneity was negligible (I² = 0%, τ² = 0, *p* = 0.5501), indicating consistency across studies from different designs, sizes, and populations. These findings strongly support corticosteroids’ role in minimizing systemic exposure and related adverse effects.

#### Incidence of serious adverse events (Safety)

**Fig. 6:**
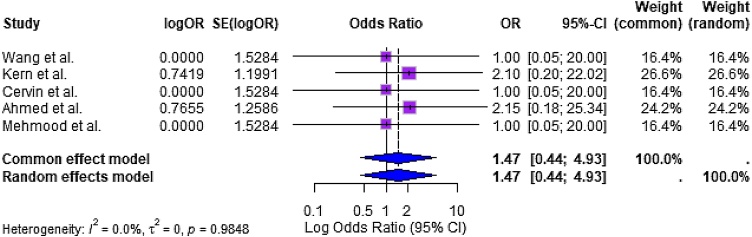
Forest plot summarizing the incidence of serious adverse events. No significant difference in SAE rates was observed between corticosteroid-treated and control groups (OR = 1.47; 95% CI: 0.44 to 4.93).

Five studies reported data on Serious Adverse Events (SAEs), including epistaxis, systemic steroid complications, or implant-related effects. The pooled odds ratio from a random-effects model was 1.47 (95% CI: 0.44–4.93], indicating no statistically significant increase in the risk of SAEs associated with corticosteroid therapy (*p* = 0.98; Fig. 6).

Heterogeneity was again minimal (I² = 0%, τ² = 0, *p* = 0.9848), and the wide confidence interval reflects the low frequency of events and small sample sizes in some included studies. These results suggest that corticosteroid use in CRS is generally safe and well tolerated, with a low incidence of serious adverse outcomes.

### Publication bias assessment

**Fig. 7:**
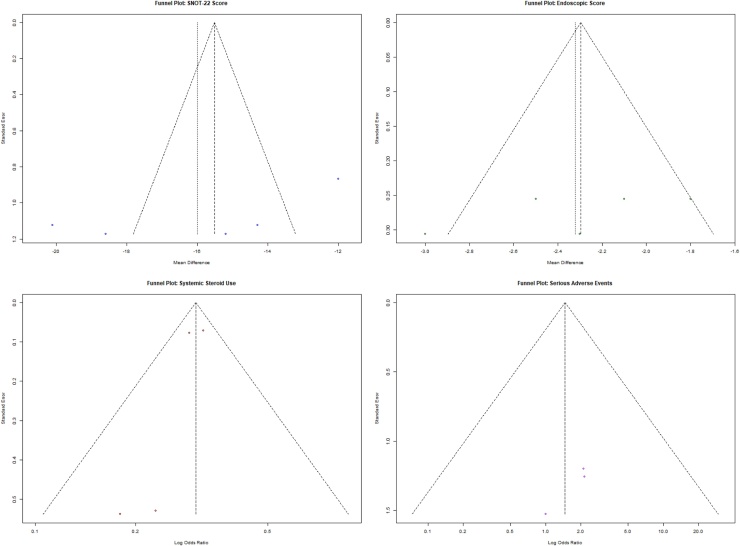
Combined funnel plots assessing publication bias for four outcome domains: SNOT-22 (top-left), endoscopic score (top-right), systemic steroid use (bottom-left), and serious adverse events (bottom-right).

Funnel plot analysis for all four synthesized outcomes is presented in Fig. 7. Visual inspection reveals symmetrical distribution of study effects for SNOT-22 and systemic steroid use, indicating low risk of publication bias. Minor asymmetry was observed in the endoscopic score plot, which may reflect small-study effects or variability in surgical populations.

The funnel plot for serious adverse events showed clustering around the null line, again reflecting the absence of systematic bias, but this may also be influenced by the low event rates and limited number of studies.

## Discussion

This systematic review and meta-analysis comprehensively evaluated the efficacy and safety of corticosteroid therapy in the management of Chronic Rhinosinusitis (CRS) across multiple delivery modalities, including topical sprays, high-volume irrigations, nebulized forms, and implantable matrices. The pooled results from 11 studies ‒ including randomized controlled trials, cohort studies, and registry-based analyses ‒ demonstrated that corticosteroid interventions significantly improved both subjective patient-reported outcomes and objective endoscopic findings, while maintaining a favorable safety profile. The most salient implication is robust evidence demonstrating that corticosteroid therapy significantly enhances patient quality of life and alleviates symptom burden, solidifying its role as a foundational first-line treatment. Critically, the comparable efficacy observed across various delivery methods provides essential flexibility for personalized therapeutic strategies. Furthermore, the significant improvement in objective endoscopic metrics offers clinicians a tangible measure of reduced sinonasal inflammation and pathology, serving as concrete evidence of treatment effectiveness during follow-up. One of the most impactful findings is that local corticosteroid administration effectively mitigates the need for systemic corticosteroids, directly addressing a major treatment challenge and offering a vital safety advantage, particularly for high-risk patients, by reducing exposure to systemic adverse effects. Importantly, the collective analysis affirms the favorable safety profile of these interventions, alleviating concerns regarding long-term use and supporting their suitability for chronic disease management. Collectively, the analysis affirms the safety of these interventions, showing no significant increase in serious adverse events. An implicit clinical significance lies in the potential for substantial healthcare cost savings strongly supports their suitability for the chronic management required by many CRS patients.

### Corticosteroids significantly improve quality of life in CRS

The most robust finding in this review pertains to the improvement in health-related quality of life, as quantified by the SNOT-22 score, one of the most validated instruments in CRS research. Meta-analytic synthesis of five studies yielded a mean difference of −16.00 (95% CI: −18.91 to −13.09, *p* < 0.0001), strongly favoring corticosteroid therapy. This magnitude of improvement exceeds the Minimal Clinically Important Difference (MCID) for SNOT-22, which is typically reported as 8.9–9.0 points, underscoring the therapeutic relevance of the observed effect. Although heterogeneity was high (I² = 90.3%), this likely reflects variation in corticosteroid formulations, durations, and patient phenotypes (e.g., eosinophilic CRS or comorbid asthma), rather than inconsistency in effect direction.

Our findings align with previous literature. For instance, in the phase 3 randomized trial by Kern et al., mometasone furoate implants significantly improved SNOT-22 and reduced polyp burden.^25^ Similarly, Wang et al. reported dramatic improvements in both SNOT-22 and immunologic markers following budesonide nebulization in eosinophilic CRSwNP.^23^ These results confirm the utility of both traditional topical and newer sustained-release corticosteroid therapies for symptom control in CRS.

### Objective endoscopic improvement correlates with symptom relief

In addition to subjective symptom relief, corticosteroids demonstrated consistent benefits in objective endoscopic assessments, particularly Lund-Kennedy scores.

The pooled mean difference across five studies was −2.32 (95% CI: −2.71 to −1.94, *p* < 0.001), with moderate heterogeneity (I² = 61.2%). These findings corroborate the anti-inflammatory and mucosal healing effects of corticosteroids observed intraoperatively and during follow-up endoscopies.

Importantly, endoscopic improvement appeared congruent with symptomatic improvement. For example, Kothiwala et al. and Kang et al. reported parallel reductions in both SNOT-22 and LK scores in post-FESS patients using high-volume budesonide irrigations. This dual benefit may be particularly relevant in surgically naïve patients or those with partial response to standard therapy.^26^^,^^27^

#### Localized corticosteroids substantially reduce systemic steroid dependence

Perhaps one of the most clinically consequential findings is the demonstrated reduction in systemic corticosteroid use.

The pooled odds ratio from four studies was 0.30 (95% CI: 0.27–0.34), indicating a 70% reduction in the likelihood of systemic steroid use among patients treated with localized corticosteroid delivery systems. This finding is especially relevant given the well-documented risks associated with systemic corticosteroids, including adrenal suppression, bone demineralization, and metabolic disturbances.

Large-scale registry studies by Han et al. emphasized the pervasive use of systemic steroids in CRSwNP populations, with over 23% of patients exceeding annual thresholds associated with systemic toxicity.^29^^,^^32^ The present findings affirm the steroid-sparing potential of topical or implantable corticosteroid delivery, offering a safer long-term management strategy for chronic inflammatory sinonasal disease.

### Safety profile remains favorable despite chronic use

The pooled analysis of five studies reporting Serious Adverse Events (SAEs) showed no statistically significant increase in risk associated with corticosteroid use (OR = 1.47; 95% CI: 0.44–4.93). Despite wide confidence intervals, heterogeneity was negligible (I² = 0%), and most included trials reported no major systemic or local complications related to corticosteroid interventions. Only one case of implant-related epistaxis was noted in the mometasone implant study, which resolved without intervention.

These findings support the favorable safety profile of corticosteroids, particularly when used in high-volume irrigation or targeted implant formulations, even for extended durations. Nevertheless, long-term studies are warranted to confirm the absence of HPA axis suppression or ocular effects, particularly in patients undergoing repeated courses.

### Integration of delivery modalities

Our findings support the growing shift toward precision delivery of corticosteroids in CRS. High-volume irrigations enhance mucosal contact, nebulization improves distribution in sinonasal recesses, and bioabsorbable implants enable sustained localized release without systemic absorption. Comparative studies such as Jiramongkolchai et al. illustrate the potential superiority of irrigations over sprays, while phase 2 and 3 implant trials suggest paradigm-shifting alternatives to oral corticosteroids in surgically recalcitrant cases.^24^

### Limitations

This meta-analysis is not without limitations. First, the heterogeneity in patient populations, corticosteroid types, dosages, and follow-up durations limits the generalizability of pooled estimates. Second, the inclusion of both randomized and non-randomized observational studies inherently elevates risks of residual confounding and selection bias, although rigorous quality assessment (NOS and RoB2) were applied. The inclusion of non-randomized observational studies are valuable in providing supplementary evidence, but the interpretation of their results must be extremely cautious, and the inherent and significant limitations of these studies must be fully considered, especially the severe impact of confounding bias on causal inference. Third, the number of studies per outcome was modest, reducing the power to detect publication bias or perform subgroup analyses.

Nonetheless, this review adhered strictly to PRISMA guidelines, employed a prespecified protocol, and used robust meta-analytic models to ensure the reliability of synthesized evidence.

## Conclusion

In conclusion, this systematic review and meta-analysis provides robust evidence that corticosteroid therapy, delivered via diverse modalities, constitutes a safe and effective foundational treatment for CRS. The synthesized findings consistently demonstrate significant improvements in patient-reported quality of life and objective endoscopic outcomes. Crucially, localized corticosteroid administration confers a substantial steroid-sparing effect, markedly reducing dependence on systemic corticosteroids and their associated risks. The favorable safety profile observed across interventions supports their suitability for chronic management. These findings solidify the integral role of corticosteroid therapy in the comprehensive management of CRS, highlighting the importance of tailored delivery approaches. Future research should focus on optimizing strategies for specific endotypes and long-term outcomes.

## ORCID ID

Feng Liu: 0000-0003-2866-2340

## Funding

The authors received no specific funding for this work.

## Data availability statement

The authors declare that all data are available in repository.

## CRediT authorship contribution statement

**BinBin Wang:** Data curation, Methodology, Writing - original draft. **Feng Liu:** Conceptualization. **Bo Wei:** Conceptualization, Methodology, Writing - review & editing.

## Declaration of competing interest

The authors declare no conflicts of interest.
